# GPs’ interest in integrated care for frail older adults and corresponding consulting and prescribing data: qualitative and quantitative analyses of the PAERPA integrated care project

**DOI:** 10.3399/BJGP.2021.0626

**Published:** 2022-10-04

**Authors:** Matthieu Calafiore, Emmanuel Chazard, Lorette Averlant, Claire Ramez, Fanny Sarrazin, Nathalie Leveque, Delphine Dambre, David Verloop, Marguerite-Marie Defebvre, Carla Di Martino, Jean-Baptiste Beuscart

**Affiliations:** Department of Family Medicine, University of Lille, Lille.; Evaluation des technologies de santé et des pratiques médicales, Centre Hospitalier Universitaire de Lille, University of Lille, Lille.; Evaluation des technologies de santé et des pratiques médicales, Centre Hospitalier Universitaire de Lille, University of Lille, Lille.; Evaluation des technologies de santé et des pratiques médicales, Centre Hospitalier Universitaire de Lille, University of Lille, Lille.; Evaluation des technologies de santé et des pratiques médicales, Centre Hospitalier Universitaire de Lille, University of Lille, Lille.; Groupe Hospitalier Territorial du Hainaut-Cambrésis, Valenciennes.; Groupe Hospitalier Territorial du Hainaut-Cambrésis, Valenciennes.; Agence Régionale de Santé Hauts-de-France, Lille.; Agence Régionale de Santé Hauts-de-France, Lille.; Evaluation des technologies de santé et des pratiques médicales, Centre Hospitalier Universitaire de Lille, University of Lille, Lille.; Evaluation des technologies de santé et des pratiques médicales, Centre Hospitalier Universitaire de Lille, University of Lille, Lille.

**Keywords:** frail older adults, preventive medicine, primary health care, general practice

## Abstract

**Background:**

Integrated care pathways can help to avoid unnecessary admissions to hospital and improve the overall quality of care for frail older patients. Although these integrated care pathways should be coordinated by GPs their level of commitment may vary.

**Aim:**

To profile GPs who had participated or had declined to participate in the Personnes Agées En Risque de Perte d’Autonomie (PAERPA) integrated care project (ICP) in the Valenciennois-Quercitain area of France between 2014 and 2019.

**Design and setting:**

A combined qualitative and quantitative analysis of GPs who were participating in or had declined to participate in the PAERPA ICP.

**Method:**

Both GPs participating in the ICP and GPs who chose not to participate in the ICP were interviewed, and then consultation and prescription profiles for these two groups were compared.

**Results:**

Some GPs were interested in the PAERPA ICP, whereas others were opposed. The 48 qualitative interviews revealed four issues that influenced participation in the PAERPA ICP: 1) awareness of issues in care of older adults and the value of collaborative work; 2) time saving; 3) task delegation; and 4) advantages of coordination. The level of interest in the ICP for frail older adults was indirectly reflected by the data on consulting and prescribing. In GPs who participated in the PAERPA ICP there was a greater proportion of older (aged ≥70 years) patients (*P*<0.05), a larger number of consultations per year (*P*<0.05), and a larger number of home visits (*P*<0.01), relative to GPs who declined to participate.

**Conclusion:**

The level of interest in the PAERPA ICP for frail older adults varied widely among GPs. These findings suggest that commitment to an integrated care pathway could be increased by customising the recruitment strategy as a function of the GP’s profile.

## INTRODUCTION

The care of frail older people is often compartmentalised by health providers and may become a series of successive but poorly coordinated assessments and procedures. Integrated care pathways seek to improve patient care by coordinating existing services and facilities. The benefits of integrated care for older patients are well established and include a reduction in potentially avoidable hospital admissions, better care at home, and a better perception of care by the patients.^1^^–^^6^

Several qualitative studies have shown that commitment by healthcare professionals is a key success factor for integrated care pathways.^7^^–^^9^ Moreover, the GP is responsible for coordinating the integrated care and has a key role in integrated care pathways for frail older adults.^10^ However, the level of GP participation in this type of pathway for older adults is variable. Although levers for and obstacles to participation have been studied, the profiles of participating and non-participating physicians do not appear to have been described.^3^^,^^7^^,^^8^^,^^11^ To the best of the authors’ knowledge, there are no published qualitative and quantitative data on this topic.

The Personnes Agées En Risque de Perte d’Autonomie (PAERPA) integrated care project (ICP) was a nationwide pilot deployed by the French Ministry of Social Affairs and Health between 2014 and 2019. It sought to provide integrated care for frail older people (aged ≥75 years) at risk of losing their independence because of medical and social factors. A personalised health plan (PHP) for integrated care was drawn up by the professionals involved in the patient’s care, with coordination by the GP. The PHP had to be agreed to in writing by all the professionals and the patient. A support platform informed healthcare professionals and patients about this new medical and social care pathway and helped them to draw up PHPs. All the PHPs were centralised and archived at a support platform dedicated to the PAERPA ICP.

The objective of the present study was to provide a qualitative and quantitative description of GPs who participated in the PAERPA ICP compared with GPs who did not.

## METHOD

### Study design

A combined qualitative and quantitative analysis of GPs who participated in the PAERPA ICP for frail older patients was undertaken. The PAERPA ICP has been implemented in 16 areas across France. The current study examined the PAERPA ICP’s deployment in the Valenciennois-Quercitain area of northern France. All GPs based in the Valenciennois-Quercitain were contacted by the PAERPA support team before and during the project’s implementation. The qualitative study was conducted between March 2017 and March 2018, and the quantitative study was conducted throughout 2018. The qualitative surveys were part of a broader study (the results of which have been published elsewhere^11^) and were reported in accordance with the Consolidated Criteria for Reporting Qualitative Research (COREQ).^12^ Twenty-nine of the COREQ checklist’s 32 items were completed.

**Table:** How this fits in

Many studies have shown that the successful implementation of integrated care requires the anticipation of barriers. At the micro-level, commitment from GPs is essential. The results of the present study show that a large proportion of GPs might not be accessible — at least at the beginning of the integrated care pathway. A more reliable strategy for including GPs at the beginning might be associated with higher acceptance and participation rates.

### Ethics

The use of healthcare data for quantitative evaluation of the PAERPA ICP was authorised by the French government (decree 2013–1090). In line with the terms of this authorisation, data were extracted from the French national health insurance system’s database by the Hauts-de-France Regional Health Authority after the study database had been registered with the French National Data Protection Commission (Commission nationale de l’informatique et des libertés [CNIL]). The GPs gave their written, informed consent to participate in the study. Audiorecordings were destroyed after transcription.

### The qualitative analysis

#### The study population

GPs who participated in the PAERPA ICP and those who declined to participate were eligible for the qualitative study. The lists of GPs were provided by the PAERPA support team. Interviewees were selected by maximum variation sampling (based on age, sex, and urban/rural location), contacted by telephone, and asked about participating in the present study. Of the GPs who participated in the PAERPA ICP, only those who had drawn up ≥3 PHPs were contacted. The recruitment process continued until no new information was generated in study interviews.

The support platform, set up specifically for the PAERPA ICP, was in regular contact with the doctors involved in PAERPA for the day-to-day PHPs. If there were two GPs with the same characteristics, the choice to contact a GP was motivated by the advice of the support platform in terms of ease of human contact. Conversely, the support platform was not in contact with the doctors who declined to participate in the PAERPA ICP. Therefore, if there were two GPs who declined to participate who had the same characteristics, the choice to contact a GP was linked to the alphabetical order of appearance in the list.

#### Data collection

Two investigators interviewed GPs who had participated in the PAERPA ICP (GP+ group) and another investigator interviewed the GPs who had declined to participate in the PAERPA ICP (GP–group). All three interviewers were residents who had received a standardised, 2-day training course in qualitative research at the University of Lille Faculty of Medicine. The interviewers did not know or had not met any of the interviewees before the study and introduced themselves by explaining that the survey was part of their MD dissertation.

The interviewers had drafted a semi-structured interview guide for each of the two groups (GP+ and GP–). The interviewers submitted and discussed their proposals for changes to the interview guides with the steering committee as the interviews progressed, through regular meetings. Approval of changes was obtained by consensus. Only the interviewer and the GP being interviewed were present during the interview, and each study participant was interviewed only once. The interview took place in the GP’s surgery and was audiorecorded. The interviews continued until sufficient data was available (that is until no new issues were identified, plus two final interviews). The studies were coordinated by a steering committee (four of the authors). Any problems or discrepancies were resolved by consensus at monthly meetings. The evolution of the interview guides between the first and the last interview is presented in Supplementary Appendix S1.

#### Analysis

Each interview was fully transcribed and anonymised. The verbatim interview was then coded independently as the interviews were conducted by two investigators and analysed according to grounded theory using a constant comparison^13^ and NVivo software (version 11).^14^ The coding allowed the emergence of categories leading to the development of a theory with the steering committee. Any disagreements were resolved by consensus with the steering committee. The steering committee and the interviewers analysed and validated the results together to describe the similarities and differences between various GPs for each identified theme.

### The quantitative study

#### The study population

GPs who had participated in the PAERPA ICP or had declined to participate in the PAERPA ICP were eligible for the quantitative study. The PAERPA support team provided the authors of the current study with the two lists of GPs and the number of PAERPA PHPs performed by each participating GP from 2015 to 2018. As a function of these data, the GPs were classified into three categories:

- those having completed ≥3 PAERPA PHPs over the 4-year period (GP+ group);
- those who had declined to participate in the PAERPA ICP (GP–group); and
- those who participated in the PAERPA ICP but had completed <3 PHPs. This category was not analysed further.

#### Data extraction

Data on the Valenciennois-Quercitain GPs’ consulting and prescribing activities between 1 January and 31 December 2018 was provided by the Hauts-de-France Regional Health Authority. The extracted data included the number of consultations, number of house calls, number of registered patients, number of patients with registered ‘chronic disease’ status, number and reimbursable value of medical lab tests, and prescriptions for medications, nursing care, and physiotherapy.

#### Data analysis

A univariate, descriptive analysis was performed first. Quantitative variables were described as the mean (standard deviation [SD]) if normally distributed or the median (interquartile range (IQR]) if not. The normality of the distribution was assessed graphically. Qualitative variables were described as the frequency (percentage). Next, the mean values of quantitative variables for the GP+ versus GP–groups of GPs were compared, using Student’s *t*-test with Welch’s correction (*n*>30). All tests were two-tailed, and the threshold for statistical significance was set to *P*<0.05. The analyses were performed using R software (version 3.5.3) and the R Studio console (version 1.1.463).

## RESULTS

### The GP population

At the time of the research (2019), the Valenciennois-Quercitain area had around 31 520 inhabitants aged ≥75 years, 350 GPs, 148 community pharmacies, 390 community nurses, five social care networks (local information and coordination centres), and several home help services for older adults.

Of the 350 GPs based in the Valenciennois-Quercitain area, 139 had completed ≥3 PAERPA PHPs (the GP+ group) and 141 GPs had declined to participate in the PAERPA ICP (the GP–group). Lastly, 70 GPs had carried out <3 PHPs and so were not analysed further.

### Results of the qualitative study

In the GP+ group, 16 of the 139 GPs were contacted. Twelve interviews were required for the collection of sufficient data. In the GP–group, 39 of the 141 GPs were contacted. Again, 12 interviews were required for the collection of sufficient data. The characteristics of the GPs interviewed and the duration of the interviews are summarised in Supplementary Table S1.

The analysis of the interviews with the GP+ and GP–groups identified four main common themes. For each theme, the GP+ and GP–groups differed diametrically in their feelings about integrated care and care for older people (Box 1). The subnodes from the coding tree are in Supplementary Figures S1 and S2.

**Box 1.** Results of the qualitative analysis of GPs’ reasons for participating or declining to participate in the PAERPA integrated care project

**GPs who participated in the PAERPA integrated care project (GP+ group)**	**GPs who declined to participate in the PAERPA integrated care project (GP–group)**
• Aware of issues in care of older adults and the value of collaborative work	• Lack of awareness of issues pertaining to care of older adults, and reluctance to work collaboratively
• Involvement in an integrated care pathway saves time	• Involvement to an integrated care pathway would be a waste of time
• Interest in delegating tasks	• Task delegation is considered as an intrusion into the physician–patient relationship
• Coordination has benefits	• The presence of a coordinator is viewed as a form of control over the physicians’ activities

*PAERPA = Personnes Agées En Risque de Perte d’Autonomie.*

The theory developed by the steering committee was based on the central role of awareness of older age care issues from which the perceptions of GP+ and GP–could be derived. The schematic diagram is provided in Supplementary Figure S3.

The participants in the GP+ group were aware of the complex issues in care of older people and considered that networking was a good way of tackling this complexity. In contrast, the members of the GP–group were not interested in care of older people and did not believe in the value of networking:

*‘… there are people who will have* [...] *skills that I don’t* [...] *I consider that they will add something to an area in which I am not competent.’*

(GP+ group, GP8, aged 58 years)

*‘It* [networking] *also gave us an overview. From a social and administrative point of view* [...] *I was very interested in that.’*

(GP+ group, GP7, aged 43 years)

*‘Geriatrics doesn’t interest me much* [...] *I’ve had it, I’m sick of it.’*

(GP–group, GP19, aged 63 years)

*‘All these networks, it’s nonsense. And anyway, they are never there when you need them.’*

(GP–group, GP24, aged 37 years)

The interviewees in the GP+ group considered that their participation in an integrated care pathway would save them time thanks to the presence of the care coordinator. Indeed, they reported they saved time because the care coordinator undertook tasks in coordination, communication, and administrative support. The members of the GP–group considered that participation would increase their workload to an unacceptable extent:

*‘I don’t think it’s feasible without a local coordinator because they coordinate the care and bind it all together* [...] *Because we can’t actually spend 2 hours coordinating with the nurse and spending 2 hours on that; it was impossible, it doesn’t fit into a GP’s schedule at the moment.’*

(GP+ group, GP6, aged 49 years)

*‘She* [the care coordinator] *made my work easier, in fact — fortunately so because it takes a lot of energy otherwise.’*

(GP+ group, GP4, aged 52 years)

*‘It’s a system that seemed very time-consuming to me. That’s what scared me.’*

(GP–group, GP15, aged 60 years)

*‘I believe that the extra work would have been impossible to do, given my workload.’*

(GP–group, GP23, aged 43 years)

The GPs in the GP+ group liked the task delegation in general and particularly with regard to the tasks taken on by the care coordinator. The members of the GP–group considered this delegation to be a form of direct intrusion into the physician–patient relationship:

*‘She* [the care coordinator] *does everything. I have to say it, she fills me in on everything. We just go over it point by point, we look together at what was targeted. But everything is filled in, I don’t have to do any extra paperwork, I don’t have an additional administrative workload.’*

(GP+ group, GP12, aged 62 years)

*‘What I liked was that the PAERPA project formalised a network that already existed.’*

(GP+ group, GP9, aged 30 years)

*‘So I didn’t wait for the PAERPA project to take care of my patients.’*

(GP–group, GP16, aged 42 years)

*‘And, in the end … the people* [patients] *who chose us as their GP have the impression that they have a rapport with us — there is intimacy, the confidentiality of the physician–patient relationship — and that this universe of intimacy is open to everyone.’*

(GP–group, GP21, aged 51 years)

The GP+ group appreciated the benefits of coordination in general and the role of the care coordinator in particular. The GP–group viewed coordination as a form of activity monitoring or a devaluation of their skills:

*‘I work on the principle that the more people we look at, the more things we see. The more people* [caregivers] *there are, the better it goes.’*

(GP+ group, GP8, aged 57 years)

*‘It’s an advantage because it’s much more practical, it centralises more things, and above all it’s very easy to contact* [the coordinator] *.’*

(GP+ group, GP12, aged 62 years)

*‘We are falling into a system, under the guise of prevention. We must not delude ourselves — they are trying to control us* [...] *I think there are organisations trying to surround us, to close us in.’*

(GP–group, GP13, aged 45 years)

*‘We are not being stifled, well, a little ... It’s guilt-driven: why didn’t he do that, why didn’t he call them? And, um, no, we’re managing alright.’*

(GP–group, GP13, aged 45 years)

### Results of the quantitative study

The characteristics of the GP+ and GP–groups are summarised and compared in Table 1. Although the two groups had similar number of registered patients, the proportion of adults aged ≥70 years was significantly higher in the GP+ group. The ratio between the number of consultations and the number of registered patients was higher in the GP+ group. Furthermore, the number of consultations per year and the number of house calls per year were higher in the GP+ group. Lastly, the number of physiotherapy procedures per registered patient, the number of prescriptions for medications and laboratory tests, as well as the number of patients registered with the status of chronic disease, were significantly higher in the GP+ group.

**Table 1.** Patient characteristics, and prescribing and consultation activities in the GP+ and GP–groups

**Characteristic**	**GP+ (*n*= 139)**	**GP– (*n*= 141)**	***P*-value**
Number of registered patients, mean (SD)	798.2 (377.3)	716.2 (399.0)	NS
Patients aged >70 years, mean (SD)	157 (95)	145 (79)	<0.05
Number of consultations per year, mean (SD)	6122.5 (3877.2)	5101.0 (3802.1)	<0.05
Number of consultations per registered patient, mean (SD)	3.29 (1.92)	2.95 (1.64)	NS
Number of house calls, median (IQR)	847 (437–1206)	592 (338–1023)	<0.01
Number of house calls per registered patient, mean (SD)	3.77 (2.54)	3.47 (2.36)	NS
Reimbursable amount of nursing care prescribed, euros, mean (SD)	90 341 (51 338)	61 287 (47 772)	<0.001
Number of nursing procedures prescribed per GP, mean (SD)	17 784 (10 354)	12 530 (9671)	<0.001
Number of nursing acts prescribed per registered patient, mean (SD)	5.19 (0.83)	4.98 (0.83)	NS
Reimbursable amount of physiotherapy prescribed, euros, mean (SD)	80 435 (42 194)	72 939 (50 462)	NS
Number of physiotherapy procedures prescribed per registered patient, mean (SD)	17.6 (0.5)	17.8 (0.5)	<0.05
Reimbursable amount of laboratory test prescribed, euros, median (IQR)	50 624 (39 980–68 443)	40 526 (26 055–58 172)	<0.01
Number of laboratory procedures per registered prescribed patient, mean (SD)	20.0 (5.0)	20.7 (5.9)	NS
Ratio between the number of consultations and the number of registered patients, median (IQR)	6.60 (5.64–7.56)	6.17 (5.26–6.77)	<0.05
Number of patients with the status of chronic disease, mean (SD)	279.7 (129.1)	211.9 (111.7)	<0.001

*GP– = GPs who declined to participate in the PAERPA integrated care project. GP+ = GPs who participated in the PAERPA integrated care project. IQR = interquartile range. NS = not significant. PAERPA = Personnes Agées En Risque de Perte d’Autonomie. PHP = personalised health plan.*

## DISCUSSION

### Summary

In the present study, the opinions and activities of GPs participating in an ICP for frail older adults versus those who declined to participate were analysed. The qualitative analysis showed that the interviewees in the GP+ and GP–groups had opposing perceptions of integrated care and markedly different levels of interest in frail older patients. The quantitative analysis showed that the level of consulting and prescribing activity for older patients was higher in the GP+ group. These findings suggest that:

- the implementation of an integrated care system should initially target GPs with an interest in integrated care and care of older people; and
- these GPs can be identified by analysing their consulting and prescribing activity.

The higher number of physiotherapy procedures, prescriptions for medications, laboratory tests per registered patient, and the higher number of patients with chronic diseases for the GP+ group suggests that these patients had more comorbidities.^15^^–^^17^

### Strengths and limitations

The present study has a number of strengths. First, GPs who declined to participate in the PAERPA ICP as well as GPs who had participated were interviewed. Second, the current study fulfilled 29 of 32 COREQ items. Third, the qualitative element was validated and coordinated by a steering committee. Fourth, the interviewers received specific interview training. Fifth, all the coding was double checked. Finally, the qualitative element is, to the best of the authors’ knowledge, the first in this field to have examined a substantive body of primary data onto GPs’ consulting and prescribing activity; this made it possible to highlight differences between the GP+ and GP–groups and identify markers of their interest in care of older people and integrated care.

This study also has some limitations. First, the data were limited to the GPs in the Valenciennois-Quercitain area. Relative to France as a whole, this area has a high proportion of single-GP surgeries. The results should therefore be extrapolated to other geographic regions or care systems with caution. Second, the GPs’ consulting and prescribing activity might be influenced by a particular type of professional practice, such as a greater willingness to care for older people. Third, GPs who had agreed to participate in the PAERPA ICP but did not do so very actively (that is with <3 PHPs) were excluded. An ‘intention-to-treat’ analysis (by reference to clinical trial designs) was therefore not performed and it is not possible to draw firm conclusions about the determinants associated with participating in an ICP. However, the study’s design made it easier to compare GPs participating in the ICP with those who declined to participate.

### Comparison with existing literature

Several qualitative studies have looked at the barriers to and levers for implementing integrated care among healthcare professionals.^4^^,^^18^ Ling *et al* conducted 213 semi-structured interviews with GPs participating in an integrated care scheme in England.^19^ They reported that the main levers for a functioning, sustainable integrated care scheme were good personal relationships between the leaders in the various organisations, the scale of the planned activity, resource availability, support for staff in new roles, and organisational and staff stability. These findings were confirmed by a recent literature review highlighting the importance of multidisciplinary team working in overall patient management.^20^ The GPs’ professional culture should evolve taking account of the collaborative approach.^11^ This new way of working, in interdisciplinarity, is currently being deployed in the multiprofessional health centres that are expanding in French primary care services. Interprofessional primary care teams involve having a team vision and sharing goals.^21^ Valentijn *et al* showed that a better understanding of the inter-relationships among the dimensions of integrated care can be achieved by a comprehensive conceptual framework that combines the concept of primary care and integrated care.^22^ The presence of a care coordinator in the coordinated care pathway is particularly appreciated by healthcare professionals.^23^ The current study shed new light on this topic by comparing GPs who participated in integrated care with those who declined to participate. The contrasting nature of the responses in the GP+ and GP–groups highlighted the GPs’ profoundly different perceptions of integrated care and frail older patients. The GP population thus appears to be very heterogeneous; GPs needs to be approached in different ways, depending on their profile.

### Implications for research and practice

The results of the quantitative element of this study suggested that a GP’s consulting and prescribing activity is a marker of their awareness of certain medical themes. Indeed, significant differences were observed between the GP+ and GP–groups with regard to 1) the number of consultations with older patients; and 2) indirect markers of morbidity (for example, the amount of nursing care prescribed). These markers might reflect the GP’s level of interest in frail older patients. It might also be possible to analyse upstream activities for other groups of patients, for example, patients with diabetes, paediatric patients, patients with psychiatric disorders, and obstetric care.

Many studies have shown that the successful implementation of integrated care requires the anticipation of barriers at the macro-, meso-, and micro-levels.^4^ At the micro-level, commitment from GPs is essential. The results showed that a large proportion of GPs might not be accessible — at least at the beginning of the integrated care pathway. A more reliable strategy for including GPs at the beginning, perhaps using, for example, indirect indicators such as prescribing activity and participation in networks, might be associated with higher acceptance and participation rates. Peer encouragement appears to be an effective lever for spreading the implementation of integrated care.^24^

These findings and the desire for change in culture and standards suggest that innovative training courses on integrated care should be promoted in France.^25^^,^^26^ These courses could be inspired by interprofessional courses that have proven effective for pharmacists^27^ and other professions. These interprofessional courses should probably be integrated into integrated care training from the outset.

In conclusion, the findings of the present study highlight major differences between GPs in the level of interest in integrated care for frail older adults. Some GPs were naturally interested in the PAERPA ICP, whereas others were strongly opposed. These differences were indirectly reflected by the data on consulting and prescribing. These findings suggest that commitment to and participation in an integrated care pathway could be increased by customising the recruitment strategy as a function of the GP’s profile.

## Data

Data for this study are not publicly available.
